# Detection of endogenous NPY release determined by novel GRAB sensor in cultured cortical neurons

**DOI:** 10.3389/fncel.2023.1221147

**Published:** 2023-07-20

**Authors:** Emma Kragelund Christensen, Ainoa Konomi-Pilkati, Joscha Rombach, Raquel Comaposada-Baro, Huan Wang, Yulong Li, Andreas Toft Sørensen

**Affiliations:** ^1^Molecular Neuropharmacology and Genetics Laboratory, Department of Neuroscience, Faculty of Health and Medical Sciences, University of Copenhagen, Copenhagen, Denmark; ^2^State Key Laboratory of Membrane Biology, Peking University School of Life Sciences, Beijing, China; ^3^PKU-IDG/McGovern Institute for Brain Research, Beijing, China

**Keywords:** NPY, GRAB sensor, biosensor, NPY release, neuropeptides, neuronal cultures, genetically encoded fluorescent sensor

## Abstract

Neuropeptide Y (NPY) is an abundantly expressed peptide in the nervous system. Its widespread distribution along with its receptors, both centrally and peripherally, indicates its broad functions in numerous biological processes. However, the low endogenous concentration and diffuse distribution of NPY make it challenging to study its actions and dynamics directly and comprehensively. Studies on the role of NPY have primarily been limited to exogenous application, transgene expression, or knock-out in biological systems, which are often combined with pharmacological probes to delineate the involvement of specific NPY receptors. Therefore, to better understand the function of NPY in time and space, direct visualization of the real-time dynamics of endogenous NPY is a valuable and desired tool. Using the first-generation and newly developed intensiometric green fluorescent G-protein-coupled NPY sensor (GRAB NPY1.0), we, for the first time, demonstrate and characterize the direct detection of endogenously released NPY in cultured cortical neurons. A dose-dependent fluorescent signal was observed upon exogenous NPY application in nearly all recorded neurons. Pharmacologically evoked neuronal activity induced a significant increase in fluorescent signal in 32% of neurons, reflecting the release of NPY, despite only 3% of all neurons containing NPY. The remaining pool of neurons expressing the sensor were either non-responsive or displayed a notable decline in the fluorescent signal. Such decline in fluorescent signal was not rescued in cortical cultures transduced with an NPY overexpression vector, where 88% of the neurons were NPY-positive. Overexpression of NPY did, however, result in sensor signals that were more readily distinguishable. This may suggest that biological factors, such as subtle changes in intracellular pH, could interfere with the fluorescent signal, and thereby underestimate the release of endogenous NPY when using this new sensor in its present configuration. However, the development of next-generation NPY GRAB sensor technology is expected soon, and will eventually enable much-wanted studies on endogenous NPY release dynamics in both cultured and intact biological systems.

## Introduction

Today, there are approximately 100 known neuropeptides. They are synthesized and released from neurons, and almost all of them bind to G-protein coupled receptors (GPCR). Not only are they key actors on both neuronal and non-neuronal cells and act in an extensive range of biological functions in the central nervous system (CNS), but they may also serve as endocrine signaling agents that can act on organs outside the CNS (Burbach, 2011; van den Pol, 2012). Moreover, expression levels of neuropeptides are altered in many disease conditions and are thought to be directly involved in several neurological conditions. Despite the existence of a variety of available techniques and tools, there are still limitations in monitoring extracellular dynamics of neurotransmitters at physiological levels with good spatial and temporal resolution, particularly when it comes to neuropeptides (Patriarchi et al., 2018; Sun et al., 2020). Techniques that can directly visualize or monitor endogenous neuropeptide function and dynamics *ex vivo* or *in vivo* are almost non-existent.

Neuropeptide Y (NPY) was discovered back in 1982 (Tatemoto et al., 1982), but the multiple biological actions of neuropeptide Y (NPY), the circumstances under which endogenous NPY is released, and its dynamics within biological systems remain largely incompletely understood. This can be attributed to the fact that NPY, along with neuropeptides in general, is challenging to study. The challenges arise due to a common set of characteristics for neuropeptides, such as low endogenous concentrations, acting on cell surface receptors over a relatively large distance, diffuse degradation patterns, a large number of isoforms (DeLaney et al., 2018), and pronounced adsorption to surfaces (Kendrick, 1990; Kristensen et al., 2015). Moreover, since neuropeptides, including NPY, positively or negatively modulate the activity of co-released neurotransmitters, they are best understood regarding their influence on other neurotransmitter systems (Hirsch and Zukowska, 2012; Nusbaum et al., 2017). Hence, the present understanding of neuropeptide dynamics is, to a large extent, extrapolated from measures of how they modulate other neurotransmitter signaling systems, and not by directly probing the neuropeptides themselves. Owing to these technical obstacles, “*the question of neuropeptide signaling has since the discovery of neuropeptides largely faded from view with a few exceptions*” to quote Nobel Prize laureate Thomas C. Südhof (Südhof, 2017).

The prevailing techniques used to determine how NPY affects synaptic transmission are electrophysiological and typically rely on observing the effect of exogenous NPY on glutamatergic transmission (El Bahh et al., 2002; Li et al., 2017). Such indirect measurement provides high temporal resolution at the expense of poor spatial resolution because it is typically limited to recording at the single-cell or local field level. Determining the effect of endogenous NPY actions is more demanding. This can be achieved by using pharmacological probes targeting NPY receptors or exploiting NPY-system knock-out models, but again the readout is indirect and typically relies on glutamatergic transmission (Lin et al., 2004; Shiozaki et al., 2020; Cattaneo et al., 2021). To visualize the release and dynamics of NPY directly, one option is to fluorescently tag the peptide. However, peptide tagging only allows the detection of transgene NPY as it is introduced by transgene gene expression (Hoogstraaten et al., 2020). An alternative technique used to investigate endogenous NPY dynamics is microdialysis, but this also exhibits limitations. Microdialysis suffers from poor temporal resolution and is spatially limited to the probe size (Kennedy, 2013), but more importantly, the adhesive nature of NPY has made this technique notably inaccurate to measure the levels of NPY (Kendrick, 1990; Lambert et al., 1994). Altogether, these technical circumstances showcase that progress in NPY research is functionally limited by the scientific tools available.

Recent advanced technologies relying on fluorescent-based imaging using biosensors, such as the G-protein-coupled receptor (GPCR) activation-based sensors (GRAB sensors), represent a compelling breakthrough, including for NPY research. GRAB sensors are genetically encoded GPCRs tethered to a confirmation-sensitive circular-permuted EGFP (cpEGFP) that enables a ligand-binding dependent change in fluorescence intensity (Nasu et al., 2021; Wang et al., 2022; Wu et al., 2022b). GRAB sensors are highly ligand-specific and display rapid activation kinetics, thereby providing excellent spatiotemporal resolution for studying neurotransmitters or neuromodulators, such as NPY (Patriarchi et al., 2018; Sun et al., 2020; Wu et al., 2022a). The GPCR used for the recently developed NPY GRAB sensor (GRAB NPY1.0) is the NPY receptor type 1 (Y1 receptor) (Wang et al., 2022). The Y1 receptor is one of the most abundant Y-receptors in the brain of humans, rats, and mice (Sperk et al., 2007; Tasan et al., 2016). One property characterizing the Y1 receptor is that it requires the full-length NPY (1–36) for activation and that it rapidly loses affinity toward truncated variants (Larhammar and Salaneck, 2004), such as NPY3-36 that also binds to Y2 and Y5 receptors (Abid et al., 2009). Hence, the GRAB NPY1.0 sensor based on the Y1 receptor scaffold is tuned for studying the release of full-length NPY.

Until now, the current GRAB NPY1.0 sensor has been characterized in HEK293T cells and rat primary cortical neurons. The sensor displays high selectivity toward NPY and not to other neuropeptides (CRF, NTS, SST, CCK, and VIP) and classical neurotransmitters (glutamate, GABA, dopamine, acetylcholine) tested. Moreover, changes in the fluorescent signal are blocked upon application of BIBO, a specific Y1 receptor antagonist (Wang et al., 2022). Since these initial characterizations were exclusively done using exogenous applied NPY, we here investigated whether the sensor is capable of detecting the release of endogenous NPY in cultured cortical neurons obtained from mice. We transduced the cultures with an adeno-associated viral (AAV) vector expressing the GRAB NPY1.0 sensor and performed live-imaging experiments. To evoke neuronal activity, we used either a pharmacological or chemical protocol. The studies were further complemented in combination with an AAV vector expressing full-length NPY as well as employing the red-shifted calcium sensor, sRGECO.

## Materials and methods

### Neuronal cell cultures

#### Culturing of glia cells for co-culture

Neurons were co-cultured on a glial monolayer. Stocks of glial cells were prepared by isolating glial cells from 1-day-old (P1) rat cortices (male and female, randomly selected). The dissociated cells were seeded in DMEM with HEPES “1965” media (Gibco, Scotland, #52100-039) containing 11% FBS, and 0.3% penicillin-streptomycin (P/S; Sigma-Aldrich, Germany, #P0781) and grown at 37°C in a humidified incubator with 10% CO_2_. When the seeded glia had reached a ∼80% confluency, the glia cells were plated on 25 mm or 18 mm coverslips coated with poly-D-lysine (Sigma-Aldrich, Germany, #2796-99-4) and laminin (Sigma-Aldrich, Germany, #L2020) and allowed to form a monolayer before seeding the neurons. If necessary, FDU (Sigma-Aldrich, Germany, #F0503) was added to the glia cultures to avoid overgrowth. The day before seeding the neurons to the glial monolayer, the media was changed to Neurobasal A media (Thermofisher, #10888022) containing 1% Glutamax (Gibco, Scotland, #35050061), 2% B-27 Plus (Thermofisher, #A3582801), and 0.1% P/S.

#### Culturing of primary neurons

Cortex was isolated from postnatal day 1–2 (P1-P2) mice (C57Bl/6, Charles River, Germany), and the meninges were removed in ice-cold dissection media containing 10% 10xHBSS (Gibco, Scotland, #14065056), 1% P/S, 1% sodium pyruvate (Gibco, Scotland, #11360070), 1% 1M HEPES (Gibco, Scotland), and 1.2% 45% glucose (Sigma-Aldrich, Germany). The cortices were cut into smaller pieces and incubated for 20–30 min at 37°C in papain solution containing 20 units/mL papain (Worthington Biochemical, #LS003126), 1mM L-cysteine (Sigma-Aldrich, Germany, #C7352), 1.9 mM CaCl2 (Sigma-Aldrich, Germany), 0.5 mM Kynurenic acid (Sigma-Aldrich, Germany, #K3375), pH adjusted to 7.4 with HCl and oxygenated with 95% O2 and 5% CO_2_. After incubation, the tissue was washed 3 times with Neurobasal A media and carefully triturated 3–5 times with a glass Pasteur pipette with a fire-polished tip. The dissociated cortical neurons were plated at a density of 100,000 or 150,000 cells/well onto the glia monolayered 18- or 25-mm coverslips, respectively, and grown in Neurobasal A media containing 1% Glutamax, 2% B-27, and 0.1% P/S. The day after seeding the neurons, half of the media was changed to remove any cell debris. Every 3–4 days, 1/4 of the media in the wells was removed and replaced with double of the removed volume of fresh neuronal media. The use of experimental animals was conducted in concordance with the Danish Research Ethical Committee for Experimental Animals (ethical permit #2017-15-0202-00092; PI: Andreas Toft Sørensen).

### Viral vectors and transduction

The following AAV vectors were used for experiments: GRAB NPY1.0 sensor (AAV2/9-hSyn-GRAB_NPY1.0; 6E+12−4.7E+13 vg/ml, 1 μL/well); sRGECO calcium sensor (AAV2/8-Ef1a-sRGECO-WPRE; Addgene plasmid #137125; RRID:Addgene_137125; 2.2E+13 vg/ml, 0.2 μL/well) (Fenno et al., 2020); NPY1-36 vector (AAV2/8-hSyn-PreproNPY-WPREpA; full-length peptide incl. CPON; produced in-house; 7.8E+12 vg/ml, 0.5 μL/well) (Soud et al., 2019). Primary cultures were transduced after 2–3 DIV and given <14 days to assure robust expression before being used for live-cell imaging.

### Immunocytochemistry

For immunocytochemistry (ICC) staining, neurons were fixed in 2% PFA for 15 min at room temperature. The coverslips were washed three times with 7.5 mM glycine (Sigma-Aldrich, Germany) in 1xPBS to block unreacted aldehydes following a single wash in 1xPBS and stored in 1xPBS at 4°C until ICC was performed. Coverslips were transferred to a humidifying chamber and washed three times with 1xPBS before being incubated in blocking buffer containing 5% goat serum, and 0.25% Triton X-100 for 30–45 min. The coverslips were then incubated with rabbit anti-NPY antibodies recognizing the C-terminal region of full-length NPY (1:1000; N9528, Sigma-Aldrich, Germany) and chicken anti-MAP2 (1:500, Ab5392, Abcam) in 5% goat serum + 0.25% Triton X-100 (Sigma-Aldrich, Germany, #93443) at 4°C overnight. The following day, the coverslips were washed four times with 1xPBS for 5 min before incubating with the secondary antibodies (1:500; goat anti-rabbit, Alexa 568 and goat-anti chicken, Alexa 488; Invitrogen) for 1 h at room temperature in 5% goat serum + 0.25% Triton X-100 excluded from light exposure. Next, the coverslips were washed three times with 1xPBS for 5 min, mounted on slides with DAPI Fluoromount-G (SouthernBiotech, #0100-20), and allowed to dry overnight. The stained coverslips were stored at 4°C excluded from light exposure.

### Fluorescent live-cell imaging

Neurons were continuously perfused with artificial cerebrospinal fluid (aCSF) [120 mM NaCl, 5 mM KCl, 2 mM CaCl2, 2 mM MgCl2, 25 mM HEPES, and 30 mM D-glucose] at pH 7.4 and 32°C for all live imaging experiments. An RC-21BDW microscope flow chamber (Warner Instruments, Hamden, CT, USA) was used to hold the coverslips and to apply a flow of different aCSF and drug buffers during imaging at 20× with a Nikon Eclipse Ti microscope connected to two EM-CCD cameras (iXon3 897, Andor). A NIS-Elements software (Nikon, Tokyo, Japan) was used for recordings. GRAB NPY1.0 and sRGECO biosensors were excited with 488 nm and 561 nm laser lines (LED sapphire laser; Coherent Inc., Santa Clara, CA, USA), respectively. For the 488 nm LED laser excitation, a 491 nm mirror, and a 475/35 nm band-pass filter were used, while a 525/50 nm band-pass filter (AHF Analysentechnik, Tübingen-Pfrondorf, Germany) was used to detect emitted light. For the 561 nm LED laser excitation, a 561 nm mirror, and a 560/40 nm band-pass filter were used, while a 630/75 nm band-pass filter (AHF Analysentechnik, Tübingen-Pfrondorf, Germany) was used to detect emitted light. The collected images were recorded either at a frame rate of 1 frame/second when recording GRAB NPY1.0 alone or 4 frames/second when the sRGECO signal was also recorded. All recordings were performed using epifluorescence microscopy.

Epifluorescence imaging was conducted under constant slow perfusion of aCSF buffers at a flow rate of approximately 1 ml/min. To induce neuronal activity, bicuculline (Bic; 50 μM; 14340, Sigma-Aldrich, Germany; dissolved in DMSO) and 4-aminopyridine (4-AP; 250 μM; 275875, Sigma-Aldrich, Germany; dissolved in Milli-Q water) was added together with aCSF, which was carefully measured after adding the compounds to confirm a pH of 7.4. Bic is a classical competitive GABA_A_ antagonist, which increases the excitatory responses by disinhibiting neuronal signaling, whereas 4-AP is a non-selective antagonist of voltage-gated potassium (Kv) channels. Potassium chloride (KCl; 60 mM; SLCG7350; Sigma-Aldrich, Germany) in aCSF was used as an alternative stimulation method. The Y1 receptor antagonist BIBO3304 (1 μM; SML2094, Sigma Aldrich, Germany; dissolved in DMSO) and NPY (NPY1-36; 0.3, 1, 30, 1000 nM; Schafer-N, Denmark; dissolved in DMSO) were used to confirming the function of the GRAB NPY1.0 sensor. 1% bovine serum albumin (BSA) was added to all NPY-containing buffers to minimize the adsorption of NPY to surfaces. Dissolved NPY were stored in low protein binding tubes (Eppendorf Protein LoBind tubes, Sigma-Aldrich, Germany). All drugs were applied as indicated in the figures.

### Data analysis and statistics

Fiji-ImageJ 1.53r software (NIH) was used for analyzing the time-lapse files from live-cell imaging of the primary cultures. Regions of interest (ROIs) were manually drawn around the neuronal somas and the plugin Time Series Analyzer V3 was used to calculate the mean intensity of the ROIs in all frames. Moreover, the intensity of two background ROIs was calculated per video. In Excel, the given ROI values were subtracted from the average value of the two background ROIs to obtain the F-values. The change in fluorescence intensity (ΔF/F0) was calculated as (F–F_baseline)/F_baseline. For videos with moving neurons, the plugin Template Matching was used to align each frame of the stack to a template frame. For all quantifications, the mean ΔF/F0 for the entire interval of the specific compound application was calculated. For the raw fluorescence images from ICC or live-cell imaging, a false color has been added for illustrative purposes. For representative images of live-cell recordings, the background was subtracted from all images, and the Image Calculator in ImageJ was used to create the final representative images of the change in fluorescence intensity. The pseudocolor images were created using the ImageJ LUT editor plugin. Identical brightness and contrast conditions were set for all images to be compared. Image scale bars were calibrated and applied in ImageJ as well. Sample sizes are indicated in the figures, and sample size calculations were not determined before the study. All GRAB NPY1.0 transduced neurons that could be identified visually by the experimenter at baseline conditions, before applying any further reagents to aCSF, were included in the analysis. A z-project was performed on the baseline frames for each recording to properly visualize and delineate a ROI around the soma of each neuron.

The ICC analysis was performed using a LSM 700 microscope to acquire 10 images from each coverslip at a 10X magnification. 3 coverslips from each condition were used for the analysis. The number of MAP2 and NPY positive neurons was counted manually in ImageJ.

Heatmaps were generated and clustering analysis was performed in R (version 4.0.4) using RStudio (version 2022.12.0). Data were grouped and averaged into time bins to combine datasets with different frame intervals. The data were combined into a matrix with rows representing neurons and columns representing bins. The matrix rows were seriated using the ARSA method in the R package seriation (version 1.4.0). Heatmaps were created from the seriated matrices using the Heatmap function from the Complexheatmap package (version 2.12.1). For Cluster analysis, the dimensionality of the matrices was reduced by principal component analysis (PCA) using the prcomp function. Clustering within the PCA space was performed using the tsclust function from the R package dtwclust (version 5.5.12) with Euclidean distances. The number of clusters was defined using the elbow method with the kmeans clustering, from the stats package (version 4.2.1).

The rest of the graphical and statistical analyses were performed using GraphPad Prism version 9 (GraphPad Software, LLC). EC50 analysis was made using the log(agonist) vs. response (three parameters) non-linear fit. Neurons presenting negative values were discarded from the EC50 analysis. One-way analysis of variance (ANOVA) followed by Tukey’s multiple comparison test, one-way ANOVA followed by Dunnett’s multiple comparisons, two-tailed *t*-test, Mann-Whitney test, and paired Wilcoxon paired *t*-test were performed. All comparisons were two-sided and statistical significance was defined as *p < 0.05, **p < 0.01, ***p < 0.001, ****p < 0.0001, ns (non-significant) *p* > 0.05.

## Results

### Imaging NPY responses in cultured cortical neurons using the GRAB NPY1.0 sensor

**FIGURE 1:**
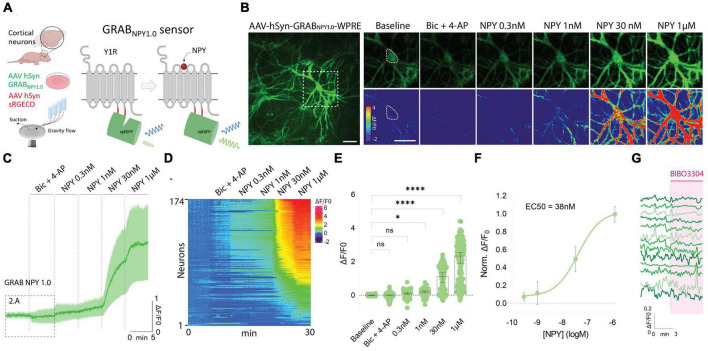
GRAB NPY1.0 sensor imaging in cultured cortical neurons during exogenous NPY application. **(A)** A schematic diagram of the basic experimental setup and principles behind the GRAB NPY1.0 sensor design. Y1R, Neuropeptide Y receptor type 1. **(B)** Left: Representative fluorescence image of GRAB NPY1.0 sensor expression during 1 μM NPY application. Scale bar, 50 μm. Right: Magnification of left image (see dotted square). The upper panels show GRAB NPY1.0 signal background subtracted during baseline, Bic + 4-AP conditions, and at different concentrations of exogenous NPY1-36. The lower panels show the corresponding scaled pseudocolor images. Scale bar, 50 μm. **(C)** Time course of GRAB NPY1.0 fluorescence presented as average (mean ± SD) ΔF/F0 trace in cortical cultures; Bic + 4-AP and different concentrations of NPY1-36 were applied where indicated. *n* = 142 neurons from 5 different coverslips. **(D)** Heatmap representation of all neurons exposed to Bic + 4-AP followed by different concentrations of applied exogenous NPY. *n* = 174 neurons from 9 different coverslips. **(E)** Quantification of mean fluorescence (ΔF/F0) at baseline, during Bic + 4-AP conditions, and different concentrations of NPY1-36. Each point represents the mean response per single neuron. *n* = 174 neurons from 9 different coverslips. Median and 95% CI are shown **(F)** normalized dose-response curve of cortical neurons expressing GRAB NPY1.0 in response to NPY. *n* = 130 neurons from 5 different coverslips. Mean and SD is shown. **(G)** Representative traces upon application of BIBO3304 to the baseline. *n* = 90 neurons from 2 different coverslips. The data in panel **(E)** were analyzed using a one-way ANOVA followed by Dunnett’s multiple comparisons. ****p < 0.0001, *p < 0.05, ns (non-significant) *p* > 0.05.

To use the GRAB NPY1.0 sensor, mouse-cultured cortical neurons were transduced with an AAV vector expressing the sensor under the control of the pan-neuronal human synapsin promoter (Figure 1A). Such a setup gives rise to exclusive neuronal expression while avoiding glial cell expression in the co-culture. The GRAB NPY1.0 sensor is composed of two basic units: the NPY Y1 receptor and the cpEGFP, serving as the ligand-sensing unit and reporter module, respectively. When the ligand binds, the EGFP fluorescent intensity increases enabling intensiometric readouts (Figure 1A). When assessing the cultures 2 weeks after transduction, sensor expression was found to be widely distributed throughout the neuronal body, including the soma and neurites (Figure 1B). All neurons whose soma could be identified based on their baseline GRAB NPY1.0 fluorescent signal were selected for the analysis, independent of changes in fluorescent signal upon subsequent manipulations. Because the release of neuropeptides from large dense-core vesicles is largely facilitated by high-frequency neuronal activity, a pharmacological combination of bicuculline (Bic) and 4-Aminopyridine (4-AP) was applied together with aCSF in the flow chamber following a baseline recording. Next, in the absence of Bic + 4-AP, exogenous NPY was applied at increasing concentrations (Figure 1B). A dose-dependent response was measured here, displaying a half-maximal effective concentration in the nanomolar range (Figures 1C–F). However, 8 out of 174 neurons expressing the GRAB NPY1.0 sensor remained unresponsive, even when exposed to 1 μM of exogenous NPY (Figures 1D, E). When applying BIBO3304, an NPY Y1 receptor antagonist that blocks the GRAB NPY1.0 sensors (Wang et al., 2022), at baseline conditions, the fluorescence signaling remained unchanged (Figure 1G), suggesting no constitutive release of endogenous NPY.

**FIGURE 2:**
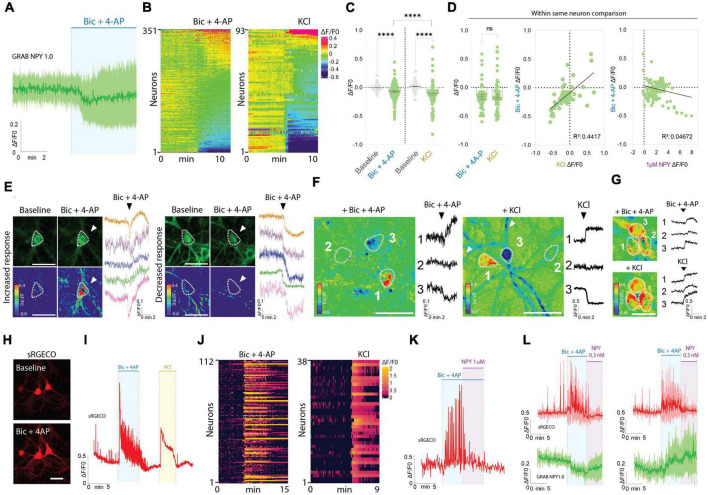
GRAB NPY1.0 sensor imaging in cortical cultures during conditions of enhanced neuronal excitability. **(A)** Magnification of the traces during Bic + 4-AP conditions shown in Figure 1C. **(B)** Heatmap representation of all neurons exposed to Bic + 4-AP, *n* = 351 from 29 different coverslips, and KCl, *n* = 93 from 5 different coverslips. **(C)** Quantification of mean fluorescent response to Bic + 4-AP and KCl stimulation of GRAB NPY1.0 expressing cortical neurons paired-compared to their corresponding baselines and non-paired between the two conditions. *n* = 351 neurons from 9 different coverslips and *n* = 93 from 5 different coverslips, respectively. **(D)** On the left, quantification and comparison between the fluorescent response (ΔF/F0) upon application of Bic + 4-AP and KCl performed in the same neuron. Middle panel, a correlation of the Bic + 4-AP and KCl responses, *n* = 56 neurons from 2 different coverslips, and right panel, a correlation between Bic + 4-AP response and NPY (1 μM) sensitivity. Median and 95% CI is shown. **(E)** Representative GRAB NPY1.0 time series images and their corresponding calibrated pseudocolored images during baseline, Bic + 4-AP conditions together with representative ΔF/F0 traces. On the left, examples of increased signal upon Bic + 4-AP application, and on the right examples of decreased fluorescence. Dotted lines indicate ROIs measured, and arrows point to the responses to Bic + 4-AP. Scale bar 50 μm. **(F)** Calibrated pseudocolored images with no background subtraction for better visualization together with ΔF/F0 traces corresponding to the outlined ROIs on the images. Showing examples of increased (1), no response (2), and decreased (3) signals in the same field of view during Bic + 4-AP (left) or KCl (right) conditions. White arrows point to the processes of the responding neurons. Scale bar 50 μm. **(G)** Calibrated pseudocolored images together with corresponding ΔF/F0 traces showing increasing responses of the same group of neurons to Bic + 4-AP or KCl. An in-between washout between the two applications was performed. The pseudocolored images are shown with no background subtraction. ΔF/F0 traces are corresponding to the outlined ROIs shown as a dotted line on the images. Scale bar 50 μm. **(H)** Representative images of sRGECO signal during baseline and Bic + 4-AP conditions. **(I)** Representative trace of sRGECO signal comparing Bic + 4-AP to KCl stimulation. **(J)** Heatmap representation of all sRGECO responses during Bic + 4-AP, *n* = 112 from 9 different coverslips, or KCl, *n* = 38 from 3 different coverslips. **(K)** Representative trace of sRGECO signal upon Bic + 4-AP application alone and together with NPY. **(L)** Time course of GRAB NPY1.0 and sRGECO fluorescence during Bic + 4-AP conditions of neurons co-expressing both sensors presented as average (mean ± SD) ΔF/F0 traces. sRGECO and GRAB NPY1.0 average responses during decreasing (left) and increasing (right) GRAB NPY1.0 signal upon Bic + 4-AP application. *n* = 69 from 8 different coverslips. The data in panel **(C)** were analyzed using a Wilcoxon paired *t*-test when comparing to baseline, and Bic + 4-AP and KCl comparison was evaluated by the Mann–Whitney test. Data in panel **(D)** was analyzed using a paired Wilcoxon paired *t*-test. ****p < 0.0001, ns (non-significant) *p* > 0.05.

Interestingly, the responses observed during Bic + 4-AP application were more diverse (Figures 2A–G). While approximately 32% of the neurons displayed an apparent increase in fluorescence intensity, suggesting the release of endogenous NPY, a fraction of neurons did not change their baseline fluorescence, while the remaining neurons surprisingly displayed a drop in fluorescent signal (Figures 2A–G). As a direct consequence, the average signal measured during Bic + 4-AP application from 351 individual neurons was statistically lower than their baseline fluorescence (Figure 2C).

Next, we tested another stimulation paradigm, applying 60 mM KCl to depolarize the neurons (Figures 2B–D). Similar to the Bic + 4-AP conditions, we observed increases and decreases in signal as well as non-responders from a total of 93 neurons recorded. This indicates that the differential responses were not exclusively related to the Bic + 4-AP application itself. Moreover, when directly comparing these two conditions, the average response was statistically lower for the KCl experiments (Figure 2C). However, a paired comparison of responses from neurons exposed to both Bic + 4-AP and KCl with an in-between washout period showed no statistical difference between the two conditions (Figure 2D). When further correlating the fluorescent intensity changes induced by these two conditions, individual neurons tended to respond similarly (Figure 2D), but this did not correlate with their response to exogenous NPY (Figure 2D). Because the GRAB NPY1.0 sensor apparently can get brighter or dimmer (Figure 2E), we additionally examined whether neighboring neurons, considering their profound interconnectivity within cultures, would display similar response patterns. Neurons within the same field of view could indeed respond as independent actors during both Bic + 4-AP and KCl conditions (Figure 2F), but tight clusters of neurons could also display a more uniform response (Figure 2G).

To gain better insight into the pharmacological protocols, that can induce a rise or decrease in fluorescent signaling (Figures 2B, E–G), we next expressed the red-shifted cytoplasmic calcium sensor sRGECO (Fenno et al., 2020) to confirm that the application of the Bic and 4-AP cocktail indeed evoked high-frequency activity in cultured cortical neurons. As expected, fluorescent spikes were substantially increased (Figures 2H–L), providing a functional visualization and confirmation of induced high-frequency neuronal activity. Furthermore, the calcium response induced by either Bic + 4-AP or KCl appeared equally potent (Figure 2I). Adding exogenous NPY to the primary cultures during continuous Bic + 4-AP perfusion, on the other hand, gave rise to a marked decrease in sRGECO fluorescent signal, reflecting suppression of neuronal activity (Figure 2K) in agreement with existing literature on NPY’s role in suppressing glutamatergic transmission (Qian et al., 1997; Acuna-Goycolea et al., 2005). Subsidiary experiments examining individual neurons co-transduced with both sensors further substantiated that elevated neuronal activity can give rise to both a raise or decrease in GRAB NPY1.0 fluorescence (Figure 2L).

Taken together, these data demonstrate that the GRAB NPY1.0 sensor is readily responding to increasing dosing of exogenous applied NPY. However, during conditions of enhanced neuronal activity, as confirmed by calcium signaling, the fluorescent response in GRAB NPY1.0 expressing neurons is not equally consistent. While a fraction of neurons increased their signaling, indicating the release of endogenous NPY, the average signaling in neuronal cultures was significantly lower as compared to their baseline fluorescence (Figure 1I).

### Detection of NPY release in cortical cultures overexpressing NPY

**FIGURE 3:**
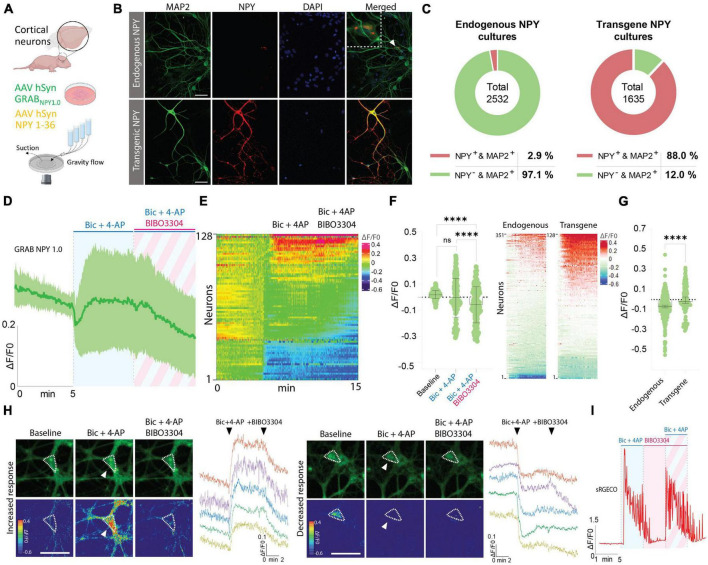
Determination of NPY-positive neurons in neuronal cultures and GRAB NPY1.0 fluorescent signaling in cultures overexpressing NPY. **(A)** Graphical representation of the experimental design. **(B)** Representative examples of ICC stainings of NPY-positive neurons in naïve and NPY transgene cultures. Scale bar 50 μm. **(C)** Percentage of NPY-positive neurons in neuronal cultures. *n* = 2,532 from 3 coverslips for endogenous NPY cultures and 1,635 from 3 coverslips for transgene NPY cultures. **(D)** Time course of GRAB NPY1.0 fluorescence presented as average (mean ± SD) ΔF/F0 trace in NPY transgene cortical cultures. **(E)** Heatmap representation of data shown in panel **(C)**. **(F)** Quantification of mean fluorescence (ΔF/F0) at baseline, Bic + 4-AP, and Bic + 4-AP + BIBO3304 application. Each circle represents the mean response per single neuron. Mean and SD is shown. **(G)** Response of NPY endogenous vs. transgene cultures to Bic + 4-AP. On the left color-simplified heatmaps showing only the Bic + 4-AP response from both conditions. Data shown in Figures 2B, 3E. On the right, average response quantification of both conditions. Data shown in Figures 2C, 3F. Median and 95% CI are shown. **(H)** Representative GRAB NPY1.0 time series images and their corresponding calibrated pseudocolored images during baseline, Bic + 4-AP, and Bic + 4-AP + BIBO3304 conditions. On the left, an example of increased signal upon Bic + 4-AP application, and on the right an example of decreased fluorescence. Dotted lines indicate ROIs measured, and arrows point to the responses to Bic + 4-AP. Scale bar 50 μm. **(I)** Representative trace of sRGECO signal during Bic + 4-AP application before and after incubation with the Y1R antagonist BIBO3304. For panels **(D–F)** *n* = 128 neurons from 6 coverslips. The data in panel **(F)** was analyzed using a one-way ANOVA followed by Tukey’s multiple comparisons test with a single pooled variance. ****p < 0.0001, ns (non-significant). Data in panel **(G)** was analyzed using a Mann–Whitney test. ****p < 0.0001.

In the next series of experiments, immunocytochemistry (ICC) was performed to validate the presence of NPY-positive neurons in naive cortical cultures, processed in parallel with cultures transduced with an AAV vector containing an expression cassette encoding full-length NPY (i.e., pre-pro-NPY) (Figures 3A, B). MAP2 staining was used as a neuronal marker and DAPI as a nuclear counterstain. Based on these stainings, 2.9% of neurons in naive cultures were found to be NPY-positive (Figures 3B, C), which is consistent with previous findings showing that an estimate of 1–2% of neurons in the cerebral cortex are NPY-positive (Aoki and Pickel, 1989). For cortical cultures transduced with the AAV vector expressing NPY, 88% of the neurons were found to be NPY-positive (Figures 3B, C).

Considering the limited number of NPY-positive neurons, including the variability in fluorescent signaling seen in previous naive cultures, we next asked whether Bic + 4-AP application could enhance transgene NPY release and, hence, be more readily detectable by the GRAB NPY1.0 sensor. Previously, transgene NPY, encoded by different viral expression systems, has been shown to be released from neurons during high-frequency neuronal activity (Sørensen et al., 2008; Hoogstraaten et al., 2020). To address the question, cultured cortical neurons were co-transduced with AAV-NPY and AAV-GRAB NPY1.0 vectors. When applying Bic + 4-AP to the cultures, a similar divergence in the sensor signaling was observed. While a portion of neurons displayed an increase others showed a decrease in fluorescence intensity (Figures 3D, E, H). Due to this variation, the overall fluorescent change was not statistically different from baseline values (Figure 3F). When further adding BIBO3304, a significant drop in fluorescence was observed (Figures 3D–F). This may suggest that transgene NPY was indeed released under the present conditions, but we cannot rule out the contribution of released endogenous NPY in addition to transgene NPY. When directly comparing the intensity changes in fluorescence between endogenous NPY and transgene NPY conditions during Bic + 4-AP application, the mean signal was significantly higher for the transgene NPY condition (Figure 3G). Also, in individual neurons displaying a noticeable increase in fluorescent signaling during Bic + 4-AP conditions and blocked by BIBO3304 application (Figure 3H), it appeared that such responses were more robust as compared to neurons responding equivalent in the naïve conditions (Figure 2E). We finally confirmed that high-frequency activity induced by Bic + 4-AP application was not *per se* affected by applying BIBO3304 to the cultures (Figure 3I).

### A subset of neurons expressing the GRAB NPY1.0 detects the release of endogenous NPY

**FIGURE 4:**
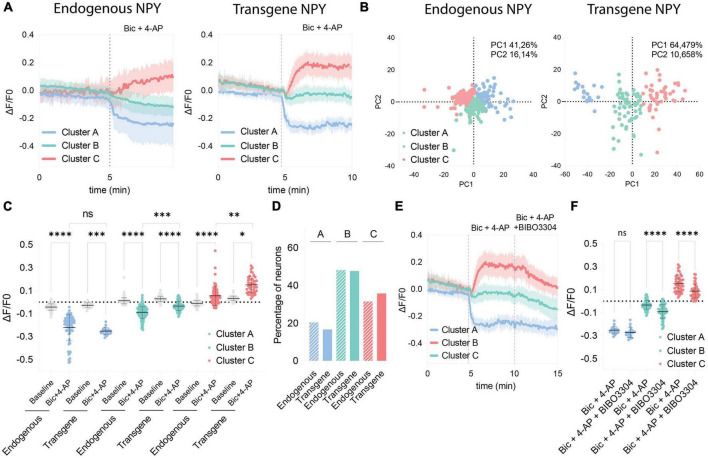
Comparison of clustered GRAB NPY1.0 signals between the endogenous and transgene NPY cultures. **(A)** Mean ΔF/F0 traces of the clustered response to Bic + 4-AP in each culture condition previously shown in Figures 1, 2. *n* = 351 and *n* = 128, respectively. The shaded area corresponds to SD. **(B)** Scatterplots from the principal component analysis showing the variance explained by the clustering for both culturing conditions. **(C)** Quantification of the average ΔF/F0 signal during baseline and upon Bic + 4-AP application for each cluster from the endogenous and transgene cultures. Median and 95% CI are shown. **(D)** Bar graph showing the percentage of neurons found in each cluster. **(E)** Mean clustered traces from transgene cultures shown in panel **(A)** together with the BIBO3304 inhibition. **(F)** Quantification of the mean signal during Bic + 4-AP and Bic + 4-AP + BIBO3304 application for each of the transgene clusters shown in panel **(E)**. Mean and SD is shown. The data in panel **(C)** were analyzed using a Kruskal–Wallis test followed by Dunnett’s multiple comparisons. The data in panel **(F)** was analyzed by one-way ANOVA with Geisser-Greenhouse correction ****p < 0.0001, ***p < 0.001, **p < 0.01, *p < 0.05, ns (non-significant) *p* > 0.05.

Because of the variability observed in the GRAB NPY1.0 sensor responses upon Bic + 4-AP conditions, we conducted a principal component analysis and subsequent clustering of the datasets. We used similar clustering parameters to cluster the datasets obtained from naïve and transduced cultures to compare the responses. Three different clusters were defined, dividing both naïve and transgene datasets into decreased (cluster A), no response (cluster B), and increased (cluster C) signals upon Bic + 4-AP application (Figure 4A). As seen from the standard deviations of the mean fluorescent traces of each cluster (Figure 4A), the clusters obtained from naïve cultures, reflecting endogenous NPY, were more overlapping than those from transgene NPY conditions. This arrangement of clusters was further visualized by plotting the two first principal components in a scatter plot (Figure 4B). From this, 57.4% of the variance could be explained for the naïve clusters, whereas 75.1% of the variance could be explained for the transgene clustering. This indicates that the data obtained from the NPY overexpression experiments were more robustly separated into defined clusters as compared to the naïve dataset. No statistical difference was observed for cluster A when comparing fluorescent signals between naïve and transgene conditions (Figure 4C). This indicates that overexpression of NPY does not affect the decreased fluorescent signaling. However, for cluster C, the signal from the transgene condition was statistically higher (Figure 4C), indicating that a higher release of NPY can be detected in the cultures overexpressing NPY. The non-responding cluster (B) also displayed a higher signal upon Bic + 4-AP application in the transgene cultures (Figure 4C), suggesting that transgene NPY release may also be detected in this cluster. The distribution of neurons among the clusters was consistent between the endogenous and transgene experiments. The majority of neurons were classified as non-responders (Cluster B; 48.12 and 47.62%, respectively), followed by increased responders (Cluster C; 31.49% and 35.71%, respectively), and decreased responders (Cluster A; 20.41% and 16.67%, respectively), which were equally distributed between experimental conditions (Figure 4D).

Finally, we assessed how the different clusters from the transgene experiment responded to BIBO3304 application during the Bic + 4-AP conditions. Cluster A was not affected by the BIBO3304 application, while signals were significantly reduced in clusters B and C (Figures 4E, F). This further confirms that the release of transgene NPY is provoked during heightened neuronal activity and hence detectable by the GRAB NPY1.0 sensor. The decrease upon BIBO3304 application seen for the “non-responders” cluster also points out that any NPY-induced signals may be masked by activity-induced quenching of fluorescence.

## Discussion

Genetically encoded fluorescent sensors are increasingly popular tools in neuroscience for investigating fundamental questions. GPCR-based biosensors are unique in their ability to directly and continuously measure the real-time dynamics of specific signaling molecules in cell cultures, tissue slices, and living animals. With a temporal resolution down to the millisecond range and a low signal-to-noise ratio, these biosensors are designed to monitor molecular dynamics at a range of physiological relevance. However, they do not provide information about the actual signaling of endogenous receptors (Jing et al., 2019; Wu et al., 2022b). As biosensors are genetically encoded, including the GRAB sensor family, they have a major advantage in being non-invasive. The coding DNA can be delivered easily into cells using viral vectors or by simple transfection, and selective spatial distribution can be achieved by using cell-type specific promoters or Cre-reporter mouse lines, making the sensors highly versatile. Consequently, biosensors have become an indispensable tool in modern brain research due to their broad use (Dong et al., 2022).

Here, we report on the usage of the GRAB NPY1.0 sensor in mouse-cultured cortical neurons and address the question: can the sensor detect the release of endogenous NPY? This sensor, recently developed in the Yulong Li laboratory, Peking University of China, is one of six neuropeptide-based GRAB sensors engineered for detecting either SST, CCK, CRF, NTS, VIP or NPY (Wang et al., 2022). While it was shown that the SST, CCK, and CRF sensors can detect endogenously released neuropeptides (Wang et al., 2022), similar information is lacking on the NPY1.0 sensor. In our setup, we tested the NPY1.0 sensor in mouse cortical cultures, where nearly 3% of the neurons were found to be NPY-positive. The average fluorescent signal robustly increased upon application of exogenous NPY, displaying an estimated EC50 value of 38 nM (Figure 1F). This responsiveness is comparable to that obtained from HEK293T cells stably expressing the NPY1.0 sensor (EC50: 40 nM). However, it is considerably higher than the EC50 value of 0.7 nM reported from rat cortical neurons transduced by a similar AAV9 vector, also harboring the human synapsin promoter used here (Wang et al., 2022). At baseline condition, perfusion with the Y1 receptors antagonist, BIBO3304, did not change the GRAB NPY1.0 output (Figure 1G). This can be interpreted in two ways; endogenous NPY is not released during these conditions, or the overexpressed sensor is not constitutively active. Intriguingly, although the wild-type NPY Y1 receptor is reported not to be constitutively active (Chee et al., 2008), the cpEGFP reporter module of the GRAB NPY sensor is inserted in the third intracellular loop of the Y1 receptor that stabilizes its inactive state. Nonetheless, our data do not suggest that the inactive state is perturbed.

To promote the release of endogenous NPY, we used either Bic + 4-AP or KCl to enhance the level of excitability. Based on sRGECO signaling, the former gave rise to high-frequency neuronal activity while the latter resulted in sustained depolarization (Figures 2H–L). We chose such protocols because NPY release occurs most prominently during high-frequency neuronal activity (Hirsch and Zukowska, 2012; Li et al., 2017), although it may also happen upon single stimulation or spontaneously (Tu et al., 2005; Li et al., 2017). During Bic + 4-AP or KCl conditions, the largest fraction of neurons did not show an increase in fluorescence. As neighboring neurons could display contrasting patterns of GRAB NPY1.0 fluorescence, this particular outcome did not appear to be attributed by any spatial factors (Figures 2F, G).

However, about 32 and 36% of the cells across the two experimental conditions, respectively, did display an increased signal intensity. This increase was significantly reduced by the Y1R antagonist, BIBO3304, which blocked the sensor (Figures 4E, F). These findings suggest that both endogenous and transgene NPY release can be measured by the sensor. However, as outlined further below, certain circumstances can affect the fluorescent signal, which may hinder accurate readouts.

Determining NPY release of individual neurons in cultures is prone to masking since an apparent quenching of the signal was not rescued in NPY overexpressing cultures despite the overall fluorescent signal being higher (Figure 3G). Hereby the actual true signal may be underestimated. The lack of signal response, even for the minor fraction of neurons exposed to high concentrations of exogenous NPY could be due to several reasons. First, and considered the most important, we selected cells based on their basal fluorescent signals. This signal was in many cases weak, implying that neurons expressing the sensor to a low amount were also included in this study. However, to provide a comprehensive characterization of the sensor, even low-expressing neurons were not excluded from the analysis. In addition, being a membrane-expressed sensor, the highest fluorescent output comes from the perimeter of the soma compared to within the center, as some membrane signals from the top and bottom of the soma can be detected since we imaged using an epifluorescence microscope. Therefore, this can affect the average signal calculated as the ROIs are drawn around the entire soma of the neuron and not only the membrane part. Moreover, the quantification of the fluorescent signal in this study was based on the mean value obtained across the entire time interval where each compound was applied and not only from the highest response. Therefore, the endogenous signal during increased neuronal activity often increased until a peak value (Figure 2E), while the mean value would be lower. Similar reflections can be made on the exogenous applied NPY, where we measured mean values, while the previous study characterizing the NPY1.0 sensor reported peak fluorescence (Wang et al., 2022).

Why does the fluorescent signal sometimes become lower in some cells? The fluorophore must be stable in the cellular microenvironment to sustain the fluorescent signal. In neuronal cultures, factors such as pH (Baird et al., 1999), oxygen, and temperature (Buckley et al., 2015; Kostyuk et al., 2019) have all been shown to affect the optical properties of the fluorophore. Particularly circular permutations, which make the fluorophore prone to acid-quenching (Baird et al., 1999). Of notice, it has been reported that excitatory synaptic activity, including seizure-like activity (Siesjö et al., 1985; Xiong et al., 2000; Raimondo et al., 2012), pharmacological glutamate receptor activation (Irwin et al., 1994), and membrane depolarizations (Hartley and Dubinsky, 1993; Zhan et al., 1998; Svichar et al., 2011), can induce transient pH variations intracellularly, but also extracellularly. Elevated K^+^ can likewise lead to an acid shift in cytosolic pH (Hartley and Dubinsky, 1993; Zhan et al., 1998; Svichar et al., 2011). We, therefore, speculate that the lack or drop in fluorescent signal could be caused by fluctuations in pH as a direct consequence of high neuronal activity.

Despite its caveats, the novel GRAB NPY1.0 represents a promising step toward advancing NPY research. Similar to genetically encoded calcium sensors, which have been optimized iteratively since the development of the first generation (Tian et al., 2012), improved variants of GRAB NPY sensors are likely to be introduced soon. For this, important elements need to be considered: a large dynamic range without compromising the sensitivity of the sensor to low ligand concentrations, selectivity, fluorescent brightness, membrane trafficking, and stabilization of the sensor. Such optimizations are a cumbersome process and mostly done by trial and error, leaving a large number of possible sequence alterations to be tested. It also needs to be determined whether overexpression of the GRAB NPY in different cell types will result in similar outcomes.

In this study, we illustrate how the development of new scientific tools can open up new scientific frontiers to be explored. Until now, it has not been possible to directly measure the endogenous release of NPY from neurons. Nevertheless, with the arrival of the novel GRAB NPY1.0 sensor, we have been able to monitor NPY release at the single-cell level. However, there is no progress without struggle. This study also pinpoints a limitation of the GRAB NPY1.0 sensor, at least in neuronal cultures, and further emphasizes the necessity of carefully characterizing new research tools before using them more broadly. To use a GRAB NPY sensor effectively in experimental settings, it may require further optimization and experimental protocols should be designed with its limitations in mind. It can be speculated whether the implementation of the GRAB NPY1.0 sensor in fiber photometry *in vivo* settings might have better potential as the output is not based on the signal from a single neuron but rather a pool of neurons. Nevertheless, the GRAB NPY1.0 sensor is a great example of the importance of research tool development in the continuous flow of science, as it brings us one step in the direction of changing the field of NPY research by presenting potential advantages over existing tools. Lastly, to quote another Nobel Laureate Sydney Brenner “*Progress depends on the interplay of techniques, discoveries, and ideas, probably in that order of decreasing importance”* (Brenner, 2002).

## Data availability statement

The original contributions presented in this study are included in this article, further inquiries can be directed to the corresponding author.

## Ethics statement

The animal study was reviewed and approved by the Danish Research Ethical Committee for Experimental Animals (ethical permit 2017-15-0202-00092; PI: AS).

## Author contributions

EC performed the experiments, completed the immunocytochemistry, did data analysis, and assisted with figure layouts. AKP contributed with neuronal culture assistance and expertise, assisted with the technical setup, did data analysis, and provided figure layouts. JR assisted with scripts used for data analysis. RC-B contributed with neuronal culture work and immunocytochemistry imaging. HW and YL developed and provided the GRAB NPY1.0 sensor. AS designed and supervised the project. EC, AKP, and AS wrote the manuscript. All authors reviewed and critically evaluated the manuscript.
